# Changes in Work Practices for Safe Use of Formaldehyde in a University-Based Anatomy Teaching and Research Facility

**DOI:** 10.3390/ijerph15092049

**Published:** 2018-09-19

**Authors:** Paul T. J. Scheepers, Martien H. F. Graumans, Gwendolyn Beckmann, Maurice van Dael, Rob B. M. Anzion, Maarten Melissen, Nicole Pinckaers, Luuk van Wel, Laurie M. A. de Werdt, Vera Gelsing, Albert van Linge

**Affiliations:** 1 Research Lab Molecular Epidemiology, Radboud Institute for Health Sciences, Radboudumc, P.O. Box 9101, 6500 HB Nijmegen, The Netherlands; martien.graumans@radboudumc.nl (M.H.F.G.); gwbeckmann@gmail.com (G.B.); Maurice.vandael@radboudumc.nl (M.v.D.); Rob.anzion@radboudumc.nl (R.B.M.A.); Maarten.melissen@digireg.nl (M.M.); nicole_pinckaers@hotmail.com (N.P.); L.vanwel@uu.nl (L.v.W.); laurie.de.werdt@arbounie.nl (L.M.A.d.W.); 2 Yacht, High Tech Campus 32, 5656 AE Eindhoven, The Netherlands; 3 Digireg, Kranestraat 37, 5961 GX Horst, The Netherlands; 4 WUR-RIKILT, Akkermaalsbos 2, 6708 WB Wageningen, The Netherlands; 5 Institute for Risk Assessment Sciences (IRAS), Utrecht University, Yalelaan 2, 3584 CM Utrecht, The Netherlands; 6 Arbo Unie, Europalaan 40, 3526 KS Utrecht, The Netherlands; 7 Department of Anatomy, Radboudumc, P.O. Box 9101, 6500 HB Nijmegen, The Netherlands; vera.gelsing@radboudumc.nl (V.G.); Albert.vanlinge@radboudumc.nl (A.v.L.)

**Keywords:** occupational hygiene, carcinogenicity, exposure assessment, risk assessment, risk management

## Abstract

Anatomy teaching and research relies on the use of formaldehyde (FA) as a preservation agent for human and animal tissues. Due to the recent classification of FA as a carcinogen, university hospitals are facing a challenge to (further) reduce exposure to FA. The aim of this study was to reduce exposure to FA in the anatomy teaching and research facility. Workers participated in the development of improved work practices, both technical and organizational solutions. Over a period of 6 years mitigating measures were introduced, including improvement of a down-flow ventilation system, introduction of local exhaust ventilation, collection of drain liquid from displayed specimens in closed containers and leak prevention. Furthermore, some organizational changes were made to reduce the number of FA peak exposures. Stationary and personal air sampling was performed in three different campaigns to assess the effect of these new work practices on inhalation exposure to FA. Samples were collected over 8 h (full shift) and 15 min (task-based) to support mitigation of exposure and improvement of work practices. Air was collected on an adsorbent coated with 2,4-dinitrophenylhydrazine (DNPH) and analyzed by HPLC-UV. Geometric mean (GM) concentrations of FA in the breathing zone over a work-shift were 123 µg/m^3^ in 2012 and 114 µg/m^3^ in 2014, exceeding the workplace standard of 150 µg/m^3^ (8 h time-weighted average, TWA) on 46% of the workdays in 2012 and 38% of the workdays in 2014. This exposure was reduced to an average of 28.8 µg/m^3^ in 2017 with an estimated probability of exceeding the OEL of 0.6%. Task-based measurements resulted in a mean peak exposures of 291 µg/m^3^ in 2012 (*n* = 19) and a mean of 272 µg/m^3^ in 2014 (*n* = 21), occasionally exceeding the standard of 500 µg/m^3^ (15 min TWA), and were reduced to a mean of 88.7 µg/m^3^ in 2017 (*n* = 12) with an estimated probability of exceeding the OEL of 1.6%.

## 1. Introduction

Most anatomy departments in hospitals and universities still rely on the use of aqueous dilutions of formaldehyde (FA) with methanol in water (formalin) for preservation and conservation of human tissues. During research and teaching, FA leads to exposure of teaching staff and students [1,2,3,4]. The laboratory staff involved in storage of the anatomical specimens for research and teaching purposes is exposed to FA and other chemicals on a daily basis. In the different work procedures such as formulation of embalming solution, transfer of (parts) of cadavers from storage and preparation of these specimens for use in teaching sessions, current workplace standards are exceeded [1,2,5].

Acute health effects have been reported in students, teachers and laboratory staff and primarily involve FA smell and sensory irritation of upper airways and eyes, sometimes in more than 50% of the exposed population [3,4]. In addition to these reversible effects, the substance is a sensitizer which can cause an allergic skin reaction [6,7] and effects on upper airways and eyes [8]. In addition to these short-term health effects, there is concern about long-term effects, including an increased risk of carcinogenicity. A recent update of the carcinogenicity classification resulted in confirmation of FA as a human carcinogen based on sufficient evidence for nasopharyngeal cancer and limited evidence for cancer of the nasal cavity and paranasal sinuses [9]. The IARC working group was not in full agreement concerning FA as a risk factor in leukaemia. Suggestions in that direction were made in a study by Hauptmann and co-workers [10] who conducted a study involving anatomy workers with peak exposures as part of embalming tasks. Kwon et al. [11] reviewed available human data and concluded that there is a causal relationship between FA exposure and both nasopharyngeal cancer and leukaemia. This is based on observed significant dose-response relationships. The occurrence of peak exposures was tentatively identified as a relevant risk factor for these tumors in occupational exposure settings.

The mechanism of toxicity is not fully understood and current findings from human biological monitoring studies were reported [12] and disputed in a reanalysis of the data [13]. Bono and co-workers [14] reported an increase of levels of leucocyte malondialdehyde-deoxyguanosine (M1-dG) adducts in pathologists exposed to levels for FA above 66 µg/m^3^. This exposure level is still below the current occupational exposure standards enforced in the US and Europe. Two studies in hospital-based occupational exposure to FA have indicated a statistical significant increase in different biomarkers of chromosomal damage. Musak and co-workers [15] reported an odds ratio of 1.7 (confidence interval 1.1–2.7) for an increased frequency of chromosomal aberrations associated with a FA exposure of pathologists from a pathological anatomy department in Central Slovakia, amounting to 320 µg/m^3^ (range 140–660 µg/m^3^), based on full shift measurements. In a second study in Portugal, Costa et al. [16] observed significantly enhanced frequencies of chromosomal aberrations and comet assay % tail DNA among anatomy pathology laboratory workers with a full-shift mean exposure of 475 ± 38 µg/m^3^, compared to control subjects.

When FA is used for disinfection purposes such as in an anatomy setting it is labelled as a ‘biocide’. This use is currently regulated in the EU because FA is classified as a chemical of high concern, following classification as a human risk factor in cancer [9] and a suspected risk factor in reproductive outcome [17,18].

More biomonitoring studies will be needed to understand the health implications of this finding, in particular to further evaluate FA exposure as a risk factor in different tumors. For reproductive risks the available data do not provide solid evidence of a classification of FA as a human reproductive risk factor [17,18] but there have been some reports that raised concern [15,19].

For this study we focused on the use of FA in a gross anatomy teaching facility that also supports research and education of students in a university hospital setting. We looked at work practices and the resulting inhalation exposure of staff and students over a period of six years. Technical and organizational changes were introduced to mitigate exposure. Both full shift and peak exposures were measured to support changes in work practices.

## 2. Materials and Methods

### 2.1. Building and Facilities

**Figure 1:**
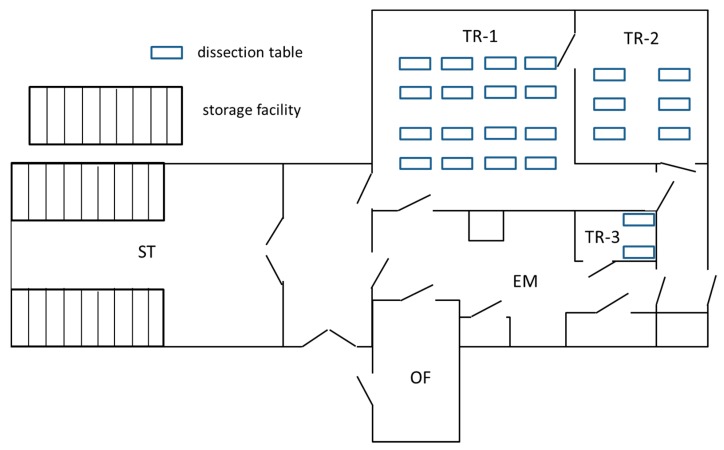
Lay-out of the Preparatorium. TR-1 = Teaching Room-1; TR-2 = Teaching Room-2; TR-3 = Teaching Room-3; EM = Embalming; ST = Storage; OF = Office.

**Figure 2:**
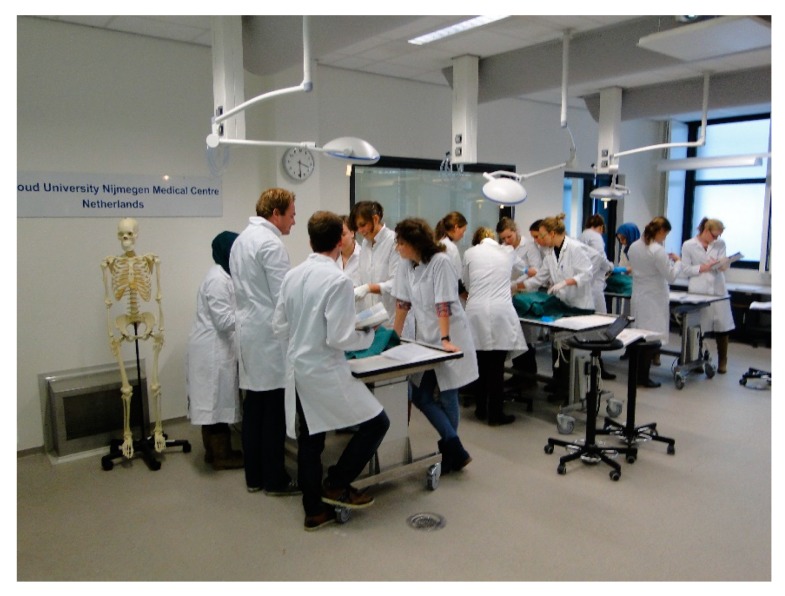
Teaching in TR-2 with air flowing into the room from the ceiling through fabric ducts and extraction vent system in the wall on the left (behind skeleton).

The anatomy facilities together called ‘Preparatorium’ are located in the basement of a preclinical building that was constructed in the 1950s. The total surface of the facility is approx. 1.000 m^2^ and consists of a storage room, an embalming room, three instruction rooms and an office (Figure 1). The storage room has a capacity of 75 storage tanks of 500 L each for storage of human tissues, referred to as ‘anatomical specimens’. These can be complete cadavers or body parts that are kept immersed in a solution of 1.9–2.2 vol % FA in tap water. The facility is also used for freezing of human remains. The latter does not involve the use of FA. Anatomical specimens are put on display to teach anatomy to medical students, used in research, and in educating the general public concerning human anatomy in an anatomy museum. In all rooms, with the exception of the embalming room, a general room ventilation system was installed, consisting of down flow ventilation with filtered and conditioned air flowing into the room from the ceiling through fabric ducts to prevent draughts (Figure 2). The air was extracted through small vent extraction units in the walls. More details are provided in Table 1.

The number of instruction tables is sixteen in training room 1 (TR-1), six in training room 2 (TR-2) and two in training room 3 (TR-3). The set-up corresponds to approximately one section table per 10 m^2^ floor surface. TR-1 and TR-2 are used for instruction as part of medical or paramedical training. TR-3 is a small room with two section tables mostly used for research and for preparation projects for the museum. The FA supply is located in the storage room and consists of a 200 L tank with a 37% FA solution, stabilized with 10% methanol. The Preparatorium employs three technicians who are responsible for all technical facilities. They maintain conservation of the collection of complete cadavers and parts (anatomical specimens), support research and training and perform all tasks related to maintenance of the technical facilities. The daily work consists of logistics, cleaning and administration and more specific tasks (described in more detail in the results section). In addition, there are some PhD students and interns who perform short-term research assignments and some student assistants who perform preparatory work for the museum. Classes are provided by instructors and student assistants, some from other universities. For characterization of the background concentrations of FA measurements on fixed locations were performed.

### 2.2. Ventilation System and Air Exchange Rate

The air exchange performance of the ventilation system was evaluated using a tracer method. At a time when the room was unoccupied the CO_2_ level was increased by the use of fire extinguishers to a level of approximately 10,000 ppm. The CO_2_ was mixed for 10 min with the air using a 50 cm diameter fan. The CO_2_ concentration was measured on a location where the overall average ventilation performance for the users of the room could be estimated. The measurements were continued over a period of 30–60 min until the CO_2_ concentration approached 1000 ppm.

### 2.3. Stationary Air Sampling

For characterization of the background concentrations of FA, measurements on fixed locations were performed. Air samples were collected using the active sampling method using the 2,4-dinitrophenylhydrazine (DNPH) method (see Section 2.6). Samples were taken at the same location every year at a height of 120 cm from the floor. In 2017 only one sample was collected in each room and some extra air samples were collected on those locations outside the anatomy facilities where the smell of FA was occasionally picked up, e.g., in an office of the anatomical museum and in a corridor close to the student’s entrance of the anatomy facility.

### 2.4. Full Shift Personal Air Sampling

Three workers employed by the Department of Anatomy were invited to participate in personal air sampling during the entire shift. In each year they were followed for three days. In addition, instructors and students were also invited to participate in the study on a voluntary basis. They would be involved as instructors or participants of the anatomy classes or as interns in research projects at the Department of Anatomy. Some students also conducted commissioned work as ‘student assistant’, e.g., on preparation projects for the museum. Instructors and students did usually not spend more time than a few hours per day. A tube with DNPH reagent coated on a solid adsorbent material was placed on their work clothes in the breathing zone. During breaks and when workers and students left the anatomy facility, the sampling equipment was left on hold in the office and mounted again upon return. All workers kept a diary with brief descriptions of the tasks they performed and specification of the duration of each task performed.

### 2.5. Task-Based Personal Air Sampling

Together with the workers, tasks were identified with potential increased exposures. Before beginning the task the sampling equipment was mounted and a measurement of 15 min was started. Most tasks took much less than 15 min to complete. In those cases, the worker or student was asked to remain in the same room to include residual exposures generated by the task in the measurement. The technical set-up was the same as for the full shift measurements (see Section 2.4).

### 2.6. FA Air Sampling and Analysis

Methanol and acetonitrile of analytical purity where purchased from Boom (Meppel, The Netherlands). Formaldehyde-2,4-dinitrophenylhydrazone (FA-2,4-DNPH) with a purity of 99.9% was obtained from Sigma-Aldrich Chemie (Zwijndrecht, The Netherlands). For calibration a known amount of this standard was weighed and diluted in acetonitrile. From this stock dilutions in acetonitril were prepared for calibration in a range of 20–20,000 µg/L. Pure acetonitrile was used as a blank. FA was collected on adsorbent tubes loaded with DNPH impregnated silica gel (SKC, Eighty Four, PA, USA) according to NIOSH method 5700. For all measurements, Buck type VSSTM-5 air sampling pumps (Buck, Orlando, FL, USA) were operated at a flow rate of 100 mL/min. This flow rate was verified before and after the measurements. The analytes were desorbed by sonication in acetonitrile. For the analysis, aliquots of this extract were injected on an Agilent 1200 Series HPLC system (Agilent Technologies, Amstelveen, The Netherlands) and separated on an Agilent Eclipse XDB-C18 (Agilent Technologies, Amstelveen, The Netherlands), 150 × 4.6 mm i.d. column with 5 µm particles. Solvent A was 100% water and Solvent B consisted of 100% methanol. Separation was achieved by gradient elution starting with 35% B, increasing to 100% B in 15 min, decreasing to 35% B in the next 3 min and stabilising during 7 min. The column was operated at a flow of 1 mL/min and 40 °C. The FA-2,4-DNPH was detected at a wavelength of 360 nm, using a UV detector (Agilent 1200 Series type VWD G1314D). The limit of quantification (LOQ) was estimated to be 1.0 µg/L. This corresponds to an air level of approx. 0.15 µg/m^3^ (see [20] for technical details).

### 2.7. Improved Work Practices

After the measurements of 2012 the workers and the management of the department started to develop some ideas for technical changes to the facility. Input for these discussions was the report from the measurement with some general recommendations and also discussion sessions in 2012 and 2014, involving the researchers and an occupational hygienist from the institutional occupational and environmental service. The results of the measurements were presented at the annual meeting of the Netherlands Society of Anatomy (www.anatomen.nl) and at annual meeting of the Netherlands Society of Occcupational Hygiene (www.arbeidshygiene.nl). During these meetings current work practices in other anatomy centers were also discussed. With the hands-on experience of the workers and with the feed-back from discussion, the team developed some technical solutions that would be expected to reduce exposure. In addition, some organisational changes were proposed to be implemented. The mitigating measures were developed along the lines of the occupational hygiene strategy (Scheepers, 2018).

### 2.8. Calculations and Statistical Analysis

The air exchange rate was calculated from the decay of CO_2_. The data were lognormally transformed and the slope factor of the linear decay was taken as an estimate for the effective air exchange. The concentrations of the FA-2,4-DNPH complex were used to calculate the air concentrations of FA. The sample volume was calculated using the lapsed sampling time and the flow rate (based on the calculated mean flow rate observed before the start and after completing the each measurement). For the figures arithmetic means and standard deviations were calculated. For the tables we calculated the geometric means and geometric standard deviations, 0.95 percentile values and the probability of exceeding the OEL was calculated using IHSTAT (American Industrial Hygiene Association, Falls Church, VA, USA). Log transformed data were evaluated in a Student’s *t*-test, using a *p* value of 0.05 as a discriminator for statistical significance.

## 3. Results

A lay-out of the Preparatorium facility is provided in Figure 1. In Table 1 an overview is presented of the room dimensions, functions and facilities. In 2012 the performance of the ventilation system was assessed and compared with the installed capacity. For the storage room an air exchange rate of 6.1 per hour was installed and achieved. For the teaching rooms the ventilation system was designed to deliver an air exchange of 15–30× the volume of the room per hour. This was achieved for TR-1 and TR-2 but not for TR-3. The technical support team discovered a malfunction that was repaired. In 2014 the performance in this room was evaluated again, still showing underperformance at 11.4 per hour (60% of the designed capacity).

Over the years several improvements of work practices were developed and implemented. Below the technical interventions will be presented (Section 3.1) and then the organizational changes that were introduced (Section 3.2). The results of measurements at fixed locations will be introduced in Section 3.3, the results of full shift personal air sampling in Section 3.4 and the results of task-based personal air sampling in Section 3.5.

### 3.1. Infrastructure and Technology

**Figure 3:**
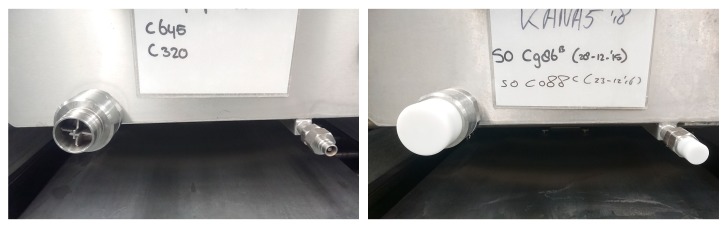
Before (**left panel**) and after (**right panel**) leak prevention of connector of storage tank by placing a cap on the open connector (T-1 in Table 2).

**Figure 4:**
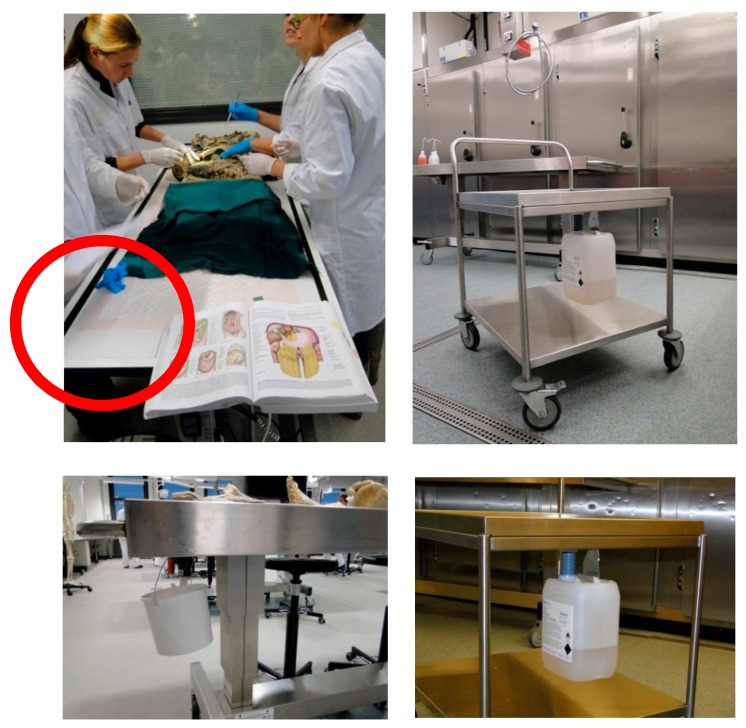
Before (**left panels**) and after (**right panels**) elimination of disposable absorbent sheets (red circle) and introduction of a containment system (instead of an open bucket) to collect leaking formalin residue (T-2 in Table 2).

**Figure 5:**
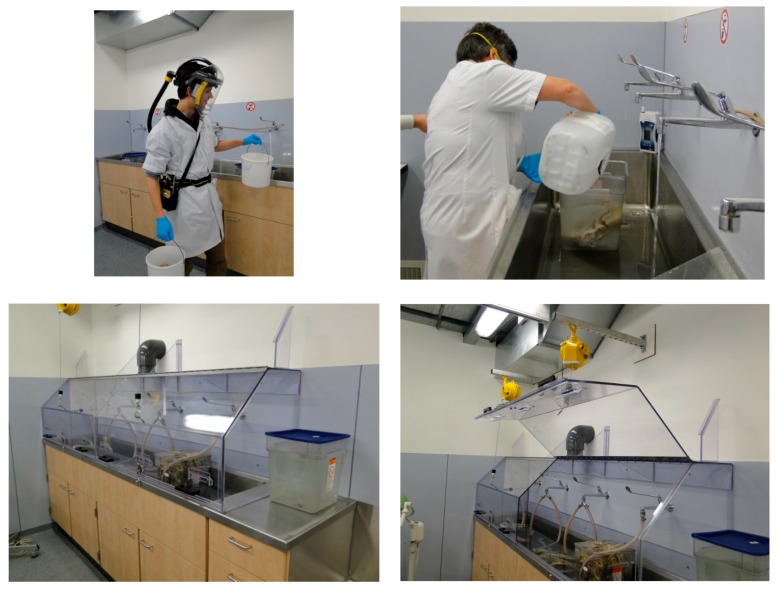
Before (**upper panels**) and after (**lower panels**) introduction of a Perspex cover with local exhaust ventilation (T-3 in Table 2).

**Figure 6:**
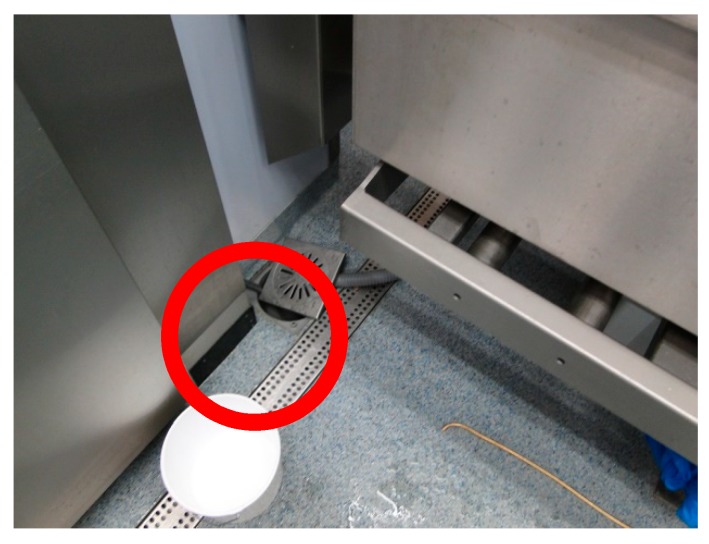
In the storage room the DFV system was blocked (red circle), preventing a free flow of air from the plenum to the extraction vent (T-4 in Table 2). Note that the grid in the floor is not for extraction of air; it is used to drain liquid spills.

**Figure 7:**
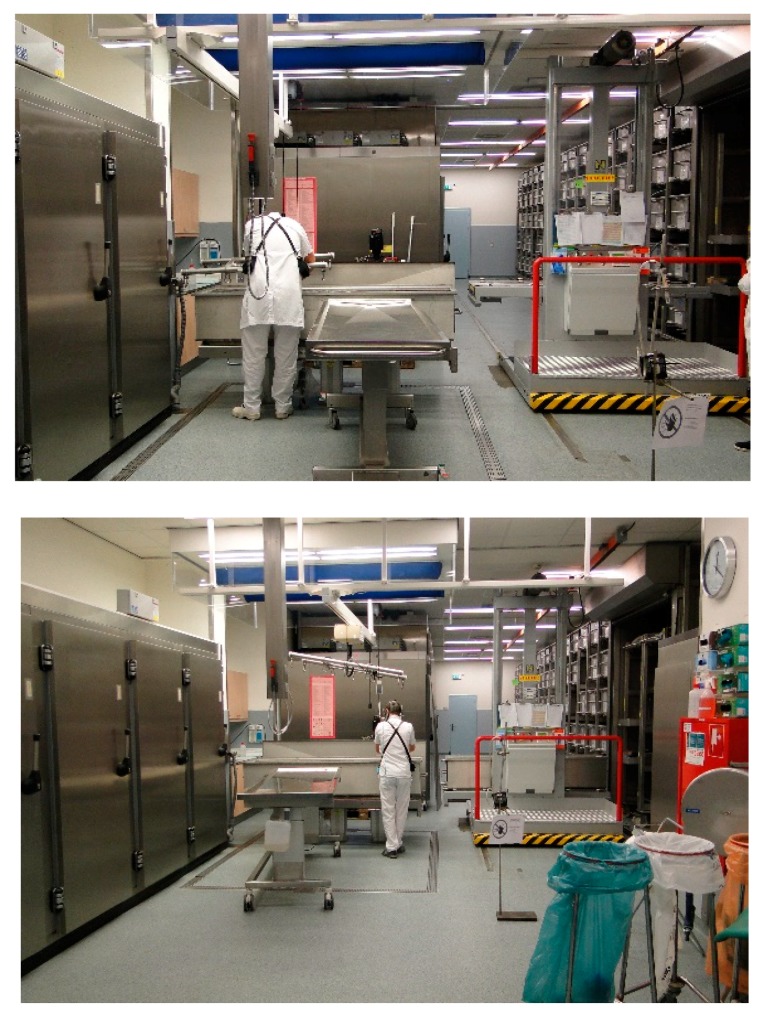
Take-out preparation from storage tank (**upper panel**) and place back preparation in storage tank (**lower panel**) (O-1 in Table 2).

An overview of the interventions for exposure mitigation is presented in Table 2 and shown in Figure 2, Figure 3, Figure 4, Figure 5, Figure 6 and Figure 7. 

The technical mitigations (T-1 to T-4) were aimed at a reduction of emissions from leakage (leak prevention, T-1, Figure 3) and emissions from the use of absorbent materials used to collect any residual draining of formalin from the anatomical specimens while they were put on display in TR-1 or TR-2. A jerry can was installed under the dissection table (T-2). So, instead of collecting the leak drained fluid in the absorbent disposable tissue and an open bucket to collect any formalin dripping from the table, (allowing the FA to evaporate from this secondary source) the drained liquid would be removed and collected in a closed containment (Figure 4). Emissions were further reduced by introducing an LEV at the workbench that was used to flush the smaller specimens (e.g. brains, extremities) prior to putting them on display on the section tables on the day of the training (T-3). This LEV system was installed in a Perspex cover that completely sealed off the process from the room and created an under pressure (Figure 5). The cover would only have to be opened for introduction or removal of specimens. During these tasks, a full face mask was used with an organic vapor and FA filter class A1 organic vapors and FA filter (6075, 3M Nederland, Delft, The Netherlands). In the storage room a down flow ventilation system was installed to introduce fresh air into the breathing zone of the worker and keep down any FA vapors from opening storage tanks and taking out specimens out of it. For this a plenum was installed above the location where an electrically powered lift was used to heave complete cadavers or body parts from the storage tank. Optimization involved a free flow of air from the plenum down to an extraction ventilation installed in the wall (Figure 6).

### 3.2. Improved Work Practices

In addition to technical improvements T-1–T-4, the work was organized differently (O-1 and O-2). The most important change of work practice was related to the system of storage of the preparation (Figure 7). Before this change, much time and effort was spent on collecting the anatomical specimens needed for a particular course from different storage tanks. In the new practice the specimens needed for a particular course were already stored in one or two tanks labeled for a specific training (O-1). An administration of the content of the storage tanks prevented the need to open more tanks than necessary: the storage tank(s) selected for a particular course would be substituted by tap water the day before, reducing the FA emissions upon opening. For this a closed system is used. After taking out the required specimens some would be taken to the embalming room for further flushing with water. The time allocated to flushing time varied from 24 h (for brains) to 48 h (for complete cadavers and for most other smaller specimens), depending on the type of preparation (O-2). According to the old practice, the specimens would be placed on the section tables (under a cover) already the day before the day with the scheduled teaching. This would cause overnight drainage of formalin residues from the specimens. According to the new practice the duration of putting the anatomical specimens on display would be kept as short as possible, e.g., from the morning just before the start of the course and removed again as soon as the training session was completed. These improved work practices would not only lead to a reduction of occupational exposure of staff but also for students who participate in the anatomy classes. A small number of measurements in the breathing zone of the instructors and students in TR-1 were able to confirm this. The mean exposure to FA in the breathing zone of instructors and students changed from 174.7 µg/m^3^ in 2012 (*n* = 5) to 102.8 µg/m^3^ in 2014 (*n* = 5) and to 30.5 µg/m^3^ in 2017 (*n* = 7).

### 3.3. Measurements at Fixed Locations

**Figure 8:**
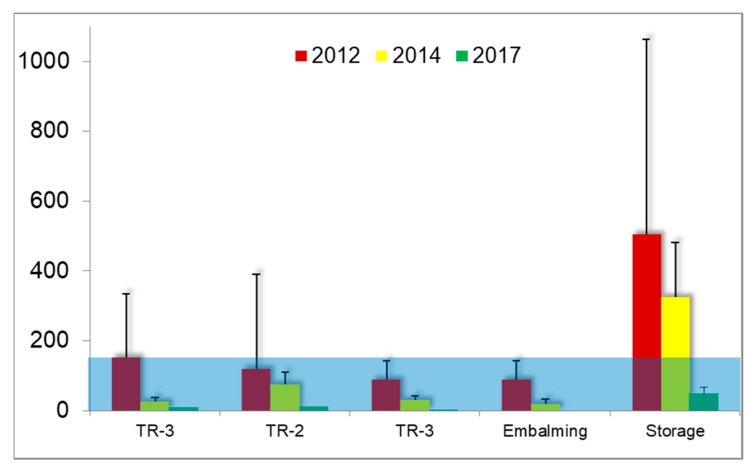
Mean ± sd FA concentrations (µg/m^3^) at fixed locations in rooms of the anatomy facility. The shaded area indicates the 8-h TWA OEL of 150 µg/m^3^ for FA in The Netherlands.

The background concentrations in each of the rooms was determined by a measurement over a full day. These measurements were taken at fixed locations and presented in Table 3 and Figure 8. 

In 2012 a background of 1.5 and 4.1 µg/m^3^ was observed in an unoccupied TR-1 with no anatomical specimens on display (empty tables). Because the educational programmes over the years were more or less similar, the reductions observed from 2012 to 2014 and 2017 in TR-1 can be attributed to a reduction in the formalin content of the specimens (increased flushing effort, O-2) and a reduction in emissions from formalin residues draining from the specimens on the disposable sheets that were used in 2012 but not any more in 2014 and 2017 (T-2). At that time jerry cans were used to collect the formalin leaking from the section tables.

In the embalming room an almost threefold reduction in average background levels was observed from 2012 to 2014. This change is attributed to the introduction of the LEV system on the work bench used to flush small specimens (T-3). No measurements were available for 2017.

In the storage room no change in the average FA concentration was observed from 2012 to 2014, indicating no effect of the optimized DFV system (T-4). In 2014 it was noted that some objects were blocking the extraction of air from the room (Figure 6). Workers were instructed to prevent any objects from blocking a free flow of air to the extraction vent opening.

One full shift measurement was performed in the office where the employees of the Preparatorium had a desk for administrative tasks (8.1 µg/m^3^). A door is connecting this room with the embalming room (Figure 1). The door is normally closed but when personnel enters or leaves the office, contaminants may enter the office from the embalming room. Staff members do not change work clothes when entering the office, making it likely that FA residues in work garments contribute to emissions of FA.

In 2017, some additional measurements were done on locations with no direct link to the ‘production floor’ of the Preparatorium. An outdoor measurement indicated that the air extracted from the Preparatorium may lead to an enhanced FA level in outdoor air of 3.8 µg/m^3^ at the location of emission (<1 m from the exhaust). This outdoor emission may explain the smell that was occasionally picked up at the office of the museum. A measurement of 4.5 µg/m^3^ showed that FA was low on the day of the measurement and this level could also be explained by other sources such as building materials [21,22]. In a corridor close to the students’ entrance of TR-1 and TR-2, a level of below 0.8 µg/m^3^ was measured. This low FA concentration may also be explained from other sources than the Preparatorium activities.

### 3.4. Full Shift Personal Air Sampling

**Figure 9:**
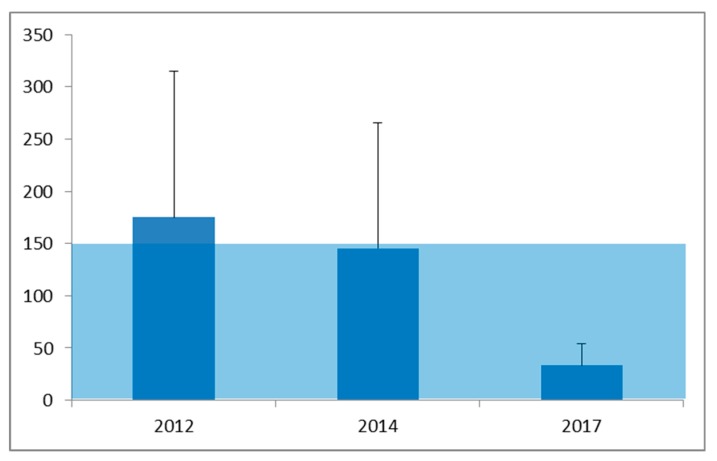
Mean ± sd FA concentrations (µg/m^3^) in the breathing zone of workers and students during a full shift. The shaded area indicates the 8-h TWA OEL of 150 µg/m^3^ for FA in The Netherlands.

The concentration of FA was measured in the breathing zone of each of the workers. In line with the definition of OELs, full shift measurements were taken (corresponding to a full work day of 8 h) as well as task-based measurements (during 15 min). An overview of the obtained results for full shift personal air sampling is presented in Table 4 by study year (Figure 9). 

In 2012 the GM for workers was very close to the 8 h TWA OEL of 150 µg/m^3^ and the 0.95 percentile value was well above this standard. In total 42.8% of the personal air samples indicated non-compliance for workers. In 2014 the worker’s exposure was similar and the overall situation was still not in compliance with the workplace standard for full shift exposure (150 µg/m^3^). Further efforts to reduce exposure were taken and resulted in a substantial decrease of exposure in 2017 with all of the measured exposures below the OEL and a probability of exceedance of this standard (based on the distribution of measurements in 2017) of 0.6%. Student assistants and instructors also participated in these measurements, being involved in teaching (TR-1 and TR-2) or in research projects (TR-3).

### 3.5. Task-Based Personal Air Sampling

**Figure 10:**
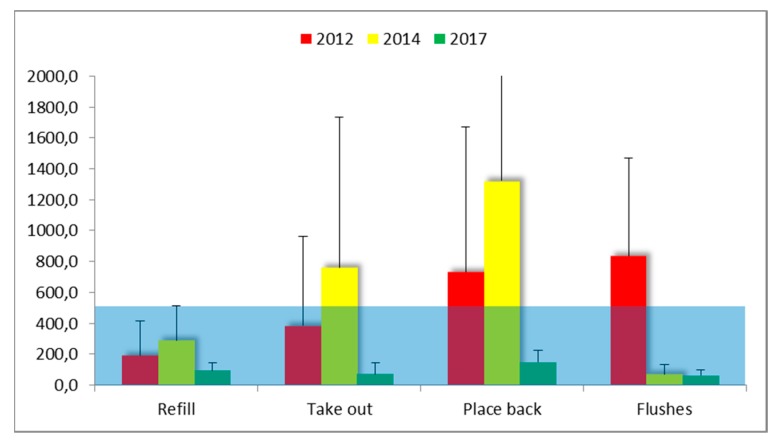
Mean ± sd FA concentrations (µg/m^3^) in the breathing zone during specific tasks. The shaded area indicates the 15-min TWA OEL of 500 µg/m^3^ for FA in the Netherlands. Refill = preparing a formalin solution from the 37% stock (storage room); Take out = searching and lifting of complete cadavers or parts from storage tanks (storage room); Place back = Immersion of complete cadavers or parts in a formalin solution in a storage tank after use (storage room). Flushes = Flushing to remove formalin from small specimens (embalming room). See Table 5 for more detailed descriptive statistics.

In Table 5 and Figure 10 an overview of the task-based measurements is provided. Overall these short-term exposures exceeded the OEL of 15 min of 500 μg/m^3^ in 31.6% of the performed tasks in 2012 and in 33.3% in 2014. In 2017 the overall performance was good with the highest exposure of 218.0 µg/m^3^ related to flushing of specimens in the embalming room.

In contrast to expectations, the handling of the 37% concentrated FA solution did not lead to the highest exposures in a timeframe of 15 min. Of the tasks measured, this task gave rise to breathing zone air concentrations that were raised above the OEL by 25.0% in 2012 and by 22.2% in 2014. In 2017 refilling remained well below the OEL but only two measurements were available so these results should be interpreted with caution. 

Flushing of small specimens with tap water resulted in the highest mean exposure of all tasks measured in 2012. With 2/3 of the measurements exceeding the OEL it was identified as a priority for improvement. Closing the system by connecting a sealed containment to an extraction ventilator, creating an under pressure, resulted in a very effective technical mitigation as shown by the tenfold reduction of the GM exposure in 2014 and 2017 (58.8 and 54.4 µg/m^3^, respectively, compared to 588.3 µg/m^3^ in 2012). This is an improvement for the worker who is performing the task but also contributes to a lower background value in the embalming room (reduction by 63% in 2014 compared to 2012).

Taking out and placing back anatomical specimens in the storage room is the task that leads to most concern of all measured tasks, especially since the evaluation of this task did not improve from 2012 to 2014. On the contrary, the measurement data indicate an increase. In part this may be related to a malfunction of the equipment which explained the highest short-term exposure value of 2769.2 µg/m^3^. Due to the improved ventilation (T-4) and new work practice (O-1) the exposure was considerably reduced to values below 50% of the OEL, compared to the task of putting the specimens back in the storage tanks in 2014. 

Near field and far field measurements conducted in 2017 showed that the far-field measurement was still more than approximately 30% of the near field measurement (68.2 versus 218 µg/m^3^). This means that bystanders would still have to wear respiratory protective equipment as well as the operator of the lift with the strap-pulley system.

A limited number of task-based measurements were done involving student assistants who performed preparation work in TR-3. In 2012 two measurements indicated task-based exposure in the breathing zone of an intern of 930 and 160 µg/m^3^. At that time the ventilation system was malfunctioning. In 2017 two additional measurements of 99.2 and 309 µg/m^3^ indicated that this task still caused enhanced exposure at a ventilation rate that was 60% of the target value.

## 4. Discussion

In 2012 the classification of FA as a risk factor for cancer in humans [9] was a game changer in many anatomy and pathology departments, regarding the use of FA for preservation purposes. Recent evaluations of published literature have confirmed a causal link between FA exposure and nasopharyngeal tumors and leukaemia [11]. Human biomonitoring studies have shown that anatomy and pathology workers with an average full-shift exposure of several hundred µg/m^3^ have a statistical significant enhanced level of chromosomal aberrations compared to hospital controls [15,16]. This biomarker has been linked to an increased cancer risk [23]. In a recent study among students in Brazil, the baseline frequency of micronucleated cells of buccal epithelial tissue was significantly increased (twofold after one month and threefold after 3.5 months) following 30–90 h of exposure to FA during human anatomy classes [24].

In the Radboudumc the anatomy teaching and research facilities had been renewed in 2009 and the exposures assessment in 2012 was the first occasion of a systematic assessment of exposure. As the outcome indicated that the conditions were not sufficiently in compliance with current OELs, the workers and the department management decided to adopt some technical improvements and changed work practices. This study describes how these changes translated in FA exposures of employees and also of instructors and students over a period of 6 years.

### 4.1. Air Sampling at Fixed Locations

FA emissions from building materials [21,22,25] offer a challenge when trying to reduce the background because of the narrow exposure margin of little more than one order of magnitude between normal indoor background levels and the OEL for a full shift. This is in particular a challenge if spills over a long time of use have caused deposits of solid polymerized formaldehyde (polyFA) that represent a persistent source of recurrent emissions because every time the floor is wetted e.g., during cleaning, some of the polymer deposit will dissolve and may contribute to FA vapors becoming airborne. In the present study we observed a background in a non-occupied teaching room which was fairly low (1.5 and 4.1 µg/m^3^) and within a range that may also be attributed to FA emissions from building materials.

In 2014, on average, a higher concentration was observed in TR-2 (69.8 µg/m^3^) compared to TR-3 (10.9 µg/m^3^). The room dimensions are different but when calculating the installed and effective ventilation per section table the infrastructure is comparable (see Table 1). A more obvious explanation for a higher background of FA in TR-2 is offered by the type of specimens used at the time of taking the air measurements. In TR-2 torsos were used that have many internal cavities that may cause draining of formalin, which resulted in a concentration of 124.6 µg/m^3^ in far field, while on the other three days concentrations of 50.3, 52.8 and 71.8 µg/m^3^ were observed at the same location [26]. In 2017, for both rooms the concentrations were low. On those days both small and large specimens were used, including complete cadavers and torsos.

In the embalming room in 2012 the average background of FA was almost 50% of the 8 h OEL with 2 out 10 measurements indicating exceedance of this workplace standard. In 2014 this situation was improved by 63% (Table 3). This improvement is attributed to introduction of an LEV system at the work bench were anatomical specimens were flushed to remove formalin (T-3).

For the storage room the observed average FA concentrations indicated exceedance of the OEL by twofold both in 2012 and 2014. At that time for all tasks performed in this room respiratory protection was required. This was feasible because only workers had access to this room and they were spending limited time for tasks specified in this report as ‘refilling’ storage tanks with formalin, ‘take out’ and ‘place back’ anatomical specimens in storage tanks after use. The high background is explained by the availability of 75 storage tanks that all represent potential sources of leakage, the availability of the 37% FA concentrate storage, the lift to take out and place back specimens from tanks and the storage cabinet for hoses that are used to fill tanks with formalin solutions. Most interventions were implemented in this room: leak prevention of storage tank (T-1), improvement of the down flow ventilation (DFV) system (T-4) and optimizing the storage system (O-1).

Within anatomy laboratories storage facilities have been identified as a location with high exposures. Higashikubo and co-workers [27] reported average pre-shift FA concentrations of 450 µg/m^3^ in Japanese anatomy facilities and reported an increase of FA exposure with installed storage capacity. Leakage of loosely sealed containers was identified as the primary source of this background. For a reduction of the background concentrations in the storage facility to a level compliant with the OEL, the required air exchange rate would have to be increased to at least 10 per hour. As long as this cannot be achieved the workers will have to wear personal protective equipment. A reduction of the 8 h TWA exposure was achieved by a restriction of the exposure duration as a result of the optimization of storage of the complete cadavers and anatomical specimens. A DFV system was installed at the location where the storage tanks are opened and the specimens are heaved from the tanks by use of the lift. An intrinsic limitation of this system is related to the principle of DFV on a location were workers and some equipment (such as the electric engine of the lift) produce heat which results in air flow in the opposite direction than the airflow of the DFV system due to convection powered by temperature differences. This effect is aggravated by the relative low room air temperature of 19 °C. Smoke testing in 2012 and 2014 showed that a downward air flow was not achieved, even in an unoccupied setting. In addition, when workers are using the facility, movements of the workers cause a turbulent airflow which further decreases the efficiency of the ventilation system.

### 4.2. Personal Air Sampling

Inhalation exposure was assessed in the breathing zone of the exposed subjects. Over the period from 2012 to 2017 this gave guidance to efforts made to reduce the number and magnitude of emission sources as determinants of inhalation exposure. Two strategies were used: The first being an overall reduction of contamination of the section tables, floors and other surfaces to try and reduce general background exposure. The 8 h TWA OEL of 150 µg/m^3^ offers good guidance to achieve an overall lower exposure level but this OEL is only 1.5 times the WHO guidance for indoor concentrations for FA [28]. The second strategy was targeting specific peak exposures arising from tasks identified by the anatomy workers as critical to FA emissions. For short-term exposures resulting from such tasks, guidance is provided by a 15 min OEL of 500 µg/m^3^. This guidance is defined as time-weighted average, which means that the value may exceed 500 µg/m^3^ for some time, as long as (within the same time interval) this exceedance is compensated with periods of low(er) exposure. Therefore, it is possible that shorter exposures (‘peaks’) of several 1000 µg/m^3^ or even higher may still occur.

Direct reading equipment with a short response time is sometimes used to analyze the tasks real-time. However, for FA these instruments have many limitations for the use in anatomy and pathology setting due the cross sensitivity with other organic compounds. False positive response may occur due to methanol which is a constituent of formalin or due to ethanol and isopropyl alcohol used in healthcare facilities for skin and surface disinfection [20]. In our study we used the DNPH method for air measurements with both 8 h and 15 min timeframes. This method also has its limitations but in active sampling it is possible to reach a sufficiently low sensitivity and good precision. Problems with high air humidity can occur [20,21,22] but is not a problem in indoor settings.

Overall, the full shift personal air sampling results indicate a gradual improvement for both workers and students from 2012 to 2014 and from 2014 to 2017 (Table 4 and Figure 9). Despite some changes of work practices, no changes were observed over the first three years (not in GM and also no in the number of measurements exceeding the OEL). This is most probably due to the situation in the storage room that was not showing a reduction in FA air concentrations (see Section 4.2). The only change that was observed is a reduction of the P_95_, bringing the concentration down from 407.9 µg/m^3^ to 252.8 µg/m^3^ but the frequency of non-compliance only decreased from 42.8% to 38.5%. For the students/instructors some improvement was observed, also in the P_95_ and in the number of exposure measurements exceeding the OEL that decreased from 60% to 40%. In the last measurements performed in 2017, all descriptors of the exposure in Table 4 indicated a substantial reduction, leading to a situation that is close to a well-controlled exposure situation. There is still some room for improvement as the GM could be further reduced from the observed overall GM of 28.8 µg/m^3^ which is close to 20% of the OEL to less than 10% of the OEL.

For the task-based measurements, the exposure of the most critical FA related work practices were (much) improved over the 6-year interval (Table 5). The highest exposures related to ‘take out’ and ‘place back’ did not show an improvement (Figure 10). For the task ‘refill’ there was no improvement either. On the contrary, the average exposure appears to be worse in 2014 compared to 2012. Because of the wide variability this is not a statistical significant increase, but it was not a reassuring finding for the workers. The high frequency of exceedance of the OEL for 15 min confirmed the necessity to keep on wearing respiratory protective equipment (full face mask with a class A1 organic vapor filter for FA, so called ‘A1 plus formaldehyde’, 3M Nederland, Delft, The Netherlands). As an extra precaution, the entrance to the room is equipped with a red flash light to indicate that the room may not be entered as long as the FA-related task is performed. The flash light is turned on manually by the worker who is performing the task.

A positive finding in 2014 was the observation of a reduction of the exposure related to the ‘flushing’ of specimens in the embalming room that was carried out using the containment with LEV. The average exposure related to this task translated in a tenfold decrease, leading to an initial frequency of exceeding the OEL from 66.7% in 2014 to 0% in 2017. The overall evaluation of task-based exposure shows a predicted probability of exceeding the 15 min OEL of 1.6%. A priority for improvement is the ventilation rate in TR-3 that was only 60% of the target value. The limited ventilation capacity would be more efficiently used when installing an LEV.

### 4.3. Strengths and Limitations of the Study

An obvious strength of this follow-up study was the worker’s participation in finding solutions to improve the technical infrastructure and (most importantly) the daily work practice. The change from 2012 to 2014 really showed one cannot rely on technological changes, only. Especially in the second stage (2014–2017) the workers themselves were able to further reduce exposure. There was good interaction and participation of the workers in identifying solutions to the critical points that were raised during the exposure studies. This process was well supported by occupational hygienists and ventilation technicians of the hospital.

Another strength of the approach was the choice for a reliable measurement methodology. The air sampling and in-house analysis of the FA-DNPH with a high standard of quality assurance based on including a complete calibration curve in each sample run.

**Figure 11:**
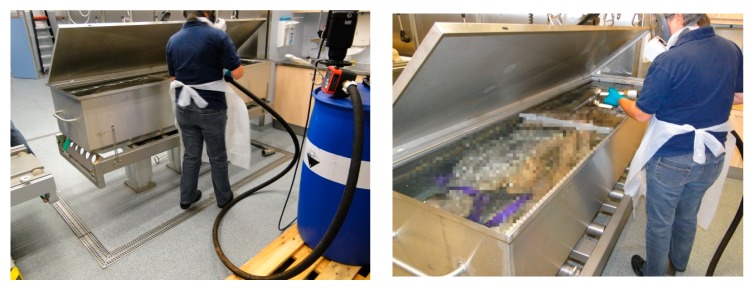
Refilling of a storage tank from the 37% FA concentrate. Note that the skin of the forearms is not protected.

The study had a focus on formaldehyde and did not consider co-exposure to methanol from the (37%/10% methanol/FA stock). Also the study did not include evaluations of skin contact and potential uptake by skin exposure (Figure 11). We did not include any short (<1 min) exposure ‘peak’ measurements; we restricted ourselves to measurements with a duration of 15 min. This was related to the lack of access to reliable measurement principle that could be used to apply real-time observations of FA exposure. Another reason for using the DNPH measurement over 15 min intervals is the definition of the established OEL for short-term exposure.

The number of task-based measurements was lower in 2017 (*n* = 12) compared to 2012 (*n* = 19) and 2014 (*n* = 21). This could lead to an underestimation of exposure measured by the number of non-compliant measurements (29 in 2012, 33 in 2014 and 0 in 2017). As the measurements have a log-normal distribution, when increasing the number of measurements the probability of finding an occasionally higher exposure increases as well. As a precaution to potential underestimation of exceedance of the OEL we calculated the probability based on the variability in the 12 task-based personal air sampling data of 2017. This resulted in a predicted probability of exceedance of the OEL of 1.6%. Regarding the suggestion that peak exposures may be of particular relevance for an increased cancer risk, future surveillance measurements are recommended.

### 4.4. Interpretation of Results in the Context of Published Literature

As was stated in one of the early occupational hygiene studies ‘Each gross anatomy facility is a unique environment’ [1]. In those days solutions of 5% were regularly used, whereas other studies proposed to use alternative formulations of preserving solutions, reducing FA contents to 0.5–0.75% [29]. In our laboratory the strategy is to keep the anatomical specimens in 1.9–2.2 vol % of FA for long-term storage but flush materials thoroughly during 24 to 48 h prior to use for research and teaching purposes.

The highest concentration of 126.6 µg/m^3^ was measured in the small section room when the thorax preparations were on display. This is in accordance with earlier reports on dissection of body cavities or deep structures when exposures were higher compared to dissection of superficial structures such as extremities [2,30,31]. Of thirteen occupational job titles reported in a national survey in Italy, the exposure of medical doctors was reported to be the highest with a geometric median of 375 µg/m^3^ with 43% of the measurements in the healthcare sector exceeding 250 µg/m^3^ [32].

Klein and co-workers suggested that planning and constructing a large-scale teaching facility with LEV installed at dissection tables leads to working conditions with consistent low exposures, below 125 µg/m^3^ [30]. Room ventilation is a general requirement but is not sufficient. Klein and co-workers observed that a two-fold increase of the air exchange rate did not result in a reduction of exposure in the breathing zone [30]. When calculating emission factors, it can be demonstrated that it is more efficient to use LEV instead of room ventilation [33,34]. The situation in the embalming room in the present study showed that a tenfold reduction in the breathing zone can be achieved by use of LEV [35]. LEV systems installed in dissection tables is current practice [36,37,38] and commercially available ducted grossing stations have been evaluated as effective systems [33], some equipped with LEV [39] or with LEV and with UV-powered photocatalytic filters to decompose FA [30].

Like in some other studies we found that both instructors and students in anatomy teaching are expected to have similar exposure levels as the workers [2]. Vohra [40] reported exposures in instructors to be higher compared to students. In a Thai gross anatomy laboratory mean (±sd) FA concentrations in the breathing zone were reported to be much higher (616 ± 116 µg/m^3^) than in the current study, which resulted in a range of symptoms including burning eyes and burning nose [41]. Other studies reported lower exposures [30].

Peak exposures are expected to be most relevant for employed staff. Students who do internships or are hired to do preparation work may also be exposed to peaks, depending on the type of research they are doing. Especially for long-term research and preparation assignments a well-ventilated room is not sufficient to prevent exposure in excess of current OELs. LEV should be installed and personal protective equipment provided along with good instructions of how to use them. In our study, removing skin from a cadaver was identified as a task associated with high exposures up to almost tenfold the OEL of 8 h. Skin incisions and subsequent release from subcutaneous adipose tissue were identified as a high emission sources, especially in embalmed female cadavers [26].

The potential of FA to cause allergic responses is well known. In animal studies repeated dermal contact with 4% solutions of FA in water, induced allergic responses in mice over a period of two weeks [42]. The allergic potency of direct skin contact is also supported by (limited) human data implicating FA as a causal factor in allergic contact dermatitis [6,7]. Therefore, in addition to prevention of inhalation, also skin protection should be or become a priority in gross anatomy facilities. In the Radboudumc facility the workers may need to consider to introduce long sleeve garment (Figure 11).

Klein et al. [31] observed a downward trend from original anatomy/pathology laboratories (1996–1999) to ‘enhanced’ original laboratories (2000–2002) and new lab (2003–2012) by use of 3 h personal measurements. The current study shows that in the same infrastructure a similar trend of exposure reduction could be achieved for fixed, full-shift and task-based measurements.

## 5. Conclusions

This study describes how workers of an anatomy department became aware of a development in the hazard classification of FA and actively participated in a long-term trajectory of changes of technical mitigation of emission sources and improved work practices. Repeated exposure assessment was used to evaluate technical changes in an air monitoring programme that reassured the workers to maintain the work practices required to keep FA emissions low, resulting in compliance with the national OEL in the Netherlands.

## Figures and Tables

**Table 1:** Technical information on the rooms available in the anatomy research and teaching facility.

Room	Size (m^2^)	Function	Facilities	Air Exchange Rate (h^−1^)		Air Exchange Per Section Table (h^−1^)	
				Design	Effective ^a^	Design	Effective ^a^
Storage	164	Storage of cadavers and specimens	Storage of human remains in 75 tanks with lifting equipment and 37% FA stock	6.1	6.3	- ^c^	- ^c^
Embalming	89	Tap water flushing	Work bench with water taps	8.8	9.0	- ^c^	-
TR-1	199	Instruction	16 dissection tables	15.3	14.2	0.96	0.89
TR-2	64	Instruction	6 dissection tables	31.3	23.7	5.2	4.0
TR-3	17	Research projects	2 dissection tables	19.5	4.6 ^b^	9.8	2.3 ^b^

^a^ Measurement using CO_2_ as a tracer (measurements performed in March 2012); ^b^ Technical malfunction (returned to 11.4 per hour after repair, leading to a capacity per table of 5.7 per hour); ^c^ -, not measured.

**Table 2:** Interventions for mitigation of exposure to FA in an anatomy facility.

Category	No.	Description	Old Work Practice	New Work Practice	Rationale	Location/Room	Figure No.
Technical	T-1	Leak prevention	Leaking of formalin by dripping of residual fluid from the tap of a storage tank	Placing a cap on the open connector to prevent leakage from taps of storage tanks	Leak reduction	Storage	2
	T-2	Elimination of disposable absorbent sheets	Specimens were placed on a disposable sheet to collect residual formalin draining from the specimens	Elimination of the disposable sheet and collection of residual formalin leakage in a closed container	Reduction of evaporation surface	Teaching	3
	T-3	Introduction of local exhaust ventilation (LEV)	Specimens are rinsed with water to remove residual formalin. For this a workbench was used with no LEV.	Placement of a Perspex containment with LEV. The containment can be opened for introduction or removal of specimens	Removal of vapors at the source	Embalming	4
	T-4	Improvement of down flow ventilation (DFV)	Create a down flow at the location where specimens are lifted from the storage tank	Improvement of ventilation equipment capacity and performance	Reduction of vapors in breathing zone	Storage	5
Organisation	O-1	Optimizing storage system ^a^	Specimens storage methods did not match with teaching programme requiring opening of many storage tanks to find the required preparation for a specific class/course	Storage of specimens needed for a specific class/course in one or a few labeled tanks to reduce on the number of tanks to be opened to retrieve the required specimens.	Reduction of the work amount	Storage	6
	O-2	Tap water flushes and reduction of exposure time	Overnight flushing of specimens by tap water	Extension of the flush time duration for specimens with a high formalin residue; reduction of the time that specimens are put on display on the dissection tables.	Removal of formalin	Embalming	7

^a^ Introduction of this new practice is still on-going. Currently this provision is in place for a few courses.

**Table 3:** Geometric mean concentrations (range) of FA (µg/m^3^) at fixed locations.

Room	2012			2014			2017		
	*n*	GM	Range	*n*	GM	Range	*n*	GM	Range
TR-1	10	80.4	49–618.3	4	21.2	7.7–38.6	1	9.0 ^d^	- ^d^
TR-2	6	10.9	2.2–672.2 ^a^	4	69.8	50.3–124.6	1	13.0 ^d^	- ^d^
TR-3	- ^b^	- ^b^	- ^b^	4	16.2	10.1–40.0	1	1.6 ^d^	- ^d^
Embalming	10	74.7	37.3–169.9	4	27.4	16.7–41.7	0	- ^d^	- ^d^
Storage	10	290.9	89.7–1506.2	4	301.7	206.5–554.0	2	62.5, 34.9 ^b,d^	- ^d^

^a^ At exhaust; ^b^ Not measured; ^c^ Two single measurements: one near field and one far field measurement, respectively; ^d^ Not calculated.

**Table 4:** Geometric mean concentrations (range) of FA (µg/m^3^) of full work-shift measurements in the breathing zone.

Year	Group	*n*	GM	P_95_	Range	Non-Compliance (%)
2012	Workers	21	123.0	407.9	17.2–519.7	42.8
	Students	5	174.7	930.0	117.0–1120	60.0
	Total	26	131.6	491.7	17.2–1120	46.2
2014	Workers	8	121.3	252.8	55.6–287.3	37.5
	Students	5	102.8	405.6	49.6–468.9	40.0
	Total	13	113.6	359.9	49.6–468.9	38.5
2017	Workers	6	26.5	61.9	10.6–71.8	0
	Students	7	30.9	68.0	19.6–80.1	0
	Total	13	28.8	75.1	10.6–80.1	0

GM = geometric mean; P_95_ = 0.95 percentile; Non-compliance = percentage of measurements exceeding the 8 h TWA OEL of 150 µg/m^3^.

**Table 5:** Geometric mean concentrations (range) of FA (µg/m^3^) of task-based 15 min measurements in the breathing zone.

Year	Description of Task	*n*	GM	P_95_	Range	Non-Compliance (%) ^b^
2012	Refill	4	104.1	460.8	35.4–510.1	25.0
	Take out	9	166.4	1423.9	58.6–1552.1	22.2
	Place back	3	345.6	1659.9	72.9–1809.6	33.3
	Flushes	3	588.3	1352.0	151.6–1395.3	66.7
	**Total**	**19**	**276.5**	**1577.8**	**58.6–1552.1**	**31.6**
2014	Refill	9	224.5	641.4	88.2–646.3	22.2
	Take out	6	418.0	2216.4	104.9–266.2	50.0
	Place back	3	947.6	2575.0	371.6–2769.2 ^a^	66.7
	Flushes	3	58.8	131.3	37.7–141.7	0.0
	**Total**	**21**	**272.0**	**2666.2**	**37.7–2769.2**	**33.3**
2017	Refill	2	-	-	61.5–128.0	0.0
	Take out	2	-	-	20.4–123.0	0.0
	Place back	3	134.6	212.6	68.2–218.0	0.0
	Flushes	3	54.4	100.5	35.8–107.0	0.0
	**Total**	**12**	**77.5**	**193.7**	**20.4–218.0**	**0.0**

^a^ Technical malfunction of the strap-pulley system; ^b^ Non-compliance = percentage of measurements exceeding the 15 min TWA OEL of 500 µg/m^3^.
